# Meta-analysis of the association of *MTHFR* polymorphisms with multiple myeloma risk

**DOI:** 10.1038/srep10735

**Published:** 2015-05-29

**Authors:** Li-Min Ma, Lin-Hai Ruan, Hai-Ping Yang

**Affiliations:** 1 Department of Hematology, The First Affiliated Hospital, Henan University of Science and Technology, Luoyang, P.R China

## Abstract

The association of methylenetetrahydrofolate reductase (*MTHFR*) polymorphisms with multiple myeloma (MM) risk has been explored, but the results remain controversial. Thus, a meta-analysis was performed to provide a comprehensively estimate. The case-control studies about *MTHFR* C677T and A1298C polymorphisms with MM risk were collected by searching PubMed, Elsevier, China National Knowledge Infrastructure and Wanfang Databases. Odds ratios (ORs) with 95% confidence intervals (CIs) were applied to assess the strength of association. Overall, no significant association was found between *MTHFR* A1298C polymorphism and MM risk under all four genetic models (AC vs. AA, OR = 0.99, 95%CI = 0.82-1.20; CC vs. AA, OR = 1.14, 95%CI = 0.77-1.68; recessive model, OR = 1.10, 95%CI = 0.76-1.59; dominant model, OR = 1.01, 95%CI = 0.84-1.22). The risk was also not significantly altered for C677T polymorphism and MM in overall comparisons (CT vs. CC, OR = 1.04, 95%CI = 0.93-1.17; TT vs. CC, OR = 1.16, 95%CI = 0.98-1.37; recessive model, OR = 1.13, 95%CI = 0.98-1.32; dominant model, OR = 1.07, 95%CI = 0.96-1.20). In subgroup analyses by ethnicity, no significant association was observed in both Caucasians and Asians. This meta-analysis suggested that *MTHFR* polymorphisms were not associated with MM risk.

Multiple myeloma (MM), the second most common hematological cancer, is a kind of plasma cells cancer characterized by bone marrow plasmacytosis and presence of monoclonal immunoglobulin^1^,^2^. MM constitutes nearly one-fifth of all hematological malignancies, and its prevalence is expected to rise in Western countries due to the population aging^3^,^4^. It has been estimated that every year, there are about 86,000 newly diagnosed patients with MM, accounting for approximately 0.8% of all new cancer cases, and 63,000 related deaths, which constitute 0.9% of all cancer deaths^3^,^5^. However, the etiology of MM remains largely unknown. In general, MM is considered as a multifactorial disease, facilitated by the interaction between various environmental and promoter factors^3^,^6^. Risk factors such as increased age, positive family history, tobacco smoking, alcohol consumption, ionizing radiation, industrial occupation, and obesity have been reported to influence the development of MM^7^,^8^. Recently, some evidence have demonstrated that genetic predisposition is also involved in MM carcinogenesis and genetic polymorphisms in candidate genes, including the immune response, DNA repair, and folate metabolism, have been found to be associated with the susceptibility to MM^9^,^10^,^11^. Moreover, increasing epidemiological studies have suggested that the promoter methylation of some candidate genes may be connected with MM pathogenesis^12^,^13^.

Methylenetetrahydrofolate reductase (MTHFR), the most critical enzyme in folate-metabolizing pathway, catalyzes the irreversible reduction of 5.10-methylenetetrahydrofolate to 5-methyltetrahydrofolate, which acts as a methyl donor for the remethylation of homocysteine into methionine^14^. Therefore, MTHFR plays an important role in the folate metabolism network and is a critical metabolic juncture in the regulation of DNA synthesis, methylation, and repair^15^,^16^. The *MTHFR* gene locates in chromosome 1p36.3^17^. Two common functional polymorphisms in the *MTHFR* gene, C677T (rs1801133) and A1298C (rs1801131), have been identified, and the variants are associated with low levels of plasma folate and significantly reduced activity of the MTHFR enzyme^18^,^19^,^20^. Previous studies have demonstrated that folate deficiency might lead to misincorporation of uracil in place of thymidine during DNA replication, resulting in DNA strand breaks and chromosomal translocation and deletion^21^,^22^. In addition, the hypomethylation of DNA may also result in activation and increased expression of proto-oncogenes, contributing to an increased prevalence of cancer^23^. Hence, individual susceptibility to cancer may be modified by some functional polymorphisms of *MTHFR* gene through the alteration of DNA synthesis and methylation. There have been several studies investigating the relationship between *MTHFR* C677T and A1298C genetic polymorphisms and MM susceptibility, but the published results are inconsistent, which may be attributed to the relatively small sample size and different ethnic background in each study^24^,^25^. Therefore, a meta-analysis was carried out to comprehensively evaluate the association of *MTHFR* C677T or A1298C polymorphisms with MM risk.

## Results

### Study characteristics

**Figure 1:**
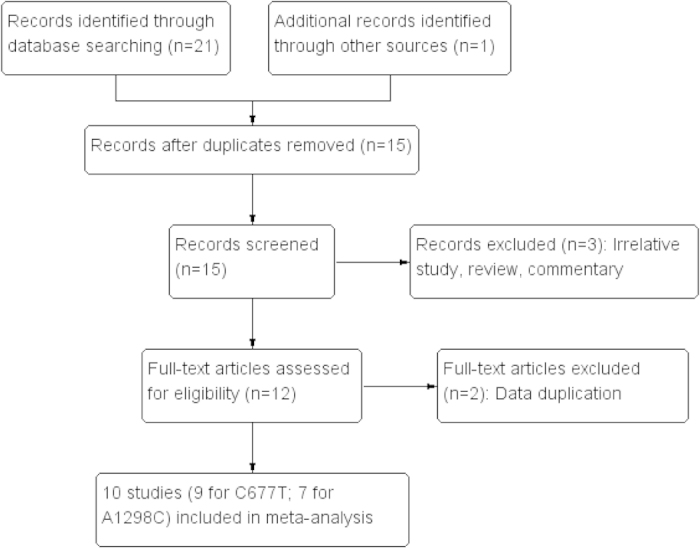
Flow diagram of study selection.

The process of study selection was showed in Fig. 1. A total of 15 potentially relevant publications were obtained through the literature search. After screening the titles, abstracts, and full-texts, five articles were excluded due to irrelevant research, review, commentary, and data duplication. Finally, a total of nine studies including 2,092 cases and 4,954 controls were included for the C677T polymorphism^24^,^25^,^26^,^27^,^28^,^29^,^30^,^31^,^32^, seven studies bearing 732 cases and 2,841 controls for the A1298C polymorphism^24^,^26^,^27^,^28^,^29^,^30^,^32^,^33^. Of these publications, there were seven studies for Caucasians^24^,^25^,^26^,^29^,^30^,^31^,^33^, and three studies for Asians^27^,^28^,^32^. When divided by the source of controls, seven studies were population-based^24^,^26^,^28^,^29^,^30^,^32^,^33^, and one was hospital-based designed^28^, respectively. The controls in the study by Martino *et al.* were selected among the general population and hospitalized subjects with diagnoses excluding cancer^32^. Seven studies with a quality score 7 or greater were considered as high quality, and three were classified into intermediate quality with score of 4-6 points. Genotypes distribution in the controls of all included studies were in consistent with HWE, except for A1298C polymorphism in Lima *et al.*^30^ Table 1 showed the detailed characteristics of included studies and the genotypes distribution of *MTHFR* C677T and A1298C polymorphisms in cases and controls was listed in Table 2.

### Results of meta-analysis

**Figure 2:**
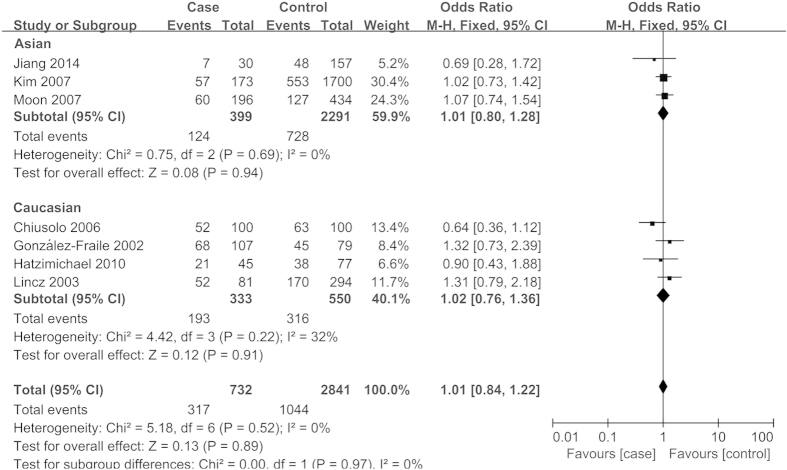
Meta-analysis for association of *MTHFR* A1298C polymorphism with MM risk (AC + CC vs. AA; stratified by ethnicity).

**Figure 3:**
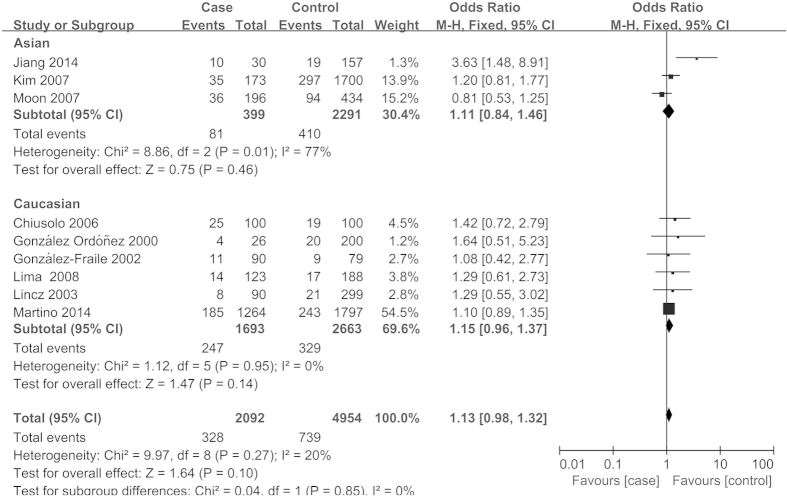
Meta-analysis for association of *MTHFR* C677T polymorphism with MM risk (TT vs. CC + CT; stratified by ethnicity).

The main results of meta-analysis and heterogeneity test were summarized in Table 3. Overall, no significant association was found between *MTHFR* A1298C polymorphism and MM risk under all four genetic models (AC vs. AA, OR = 0.99, 95%CI = 0.82-1.20, *P* = 0.92; CC vs. AA, OR = 1.14, 95%CI = 0.77-1.68, *P* = 0.51; CC vs. AA + AC, OR = 1.10, 95%CI = 0.76-1.59, *P* = 0.62; AC + CC vs. AA, OR = 1.01, 95%CI = 0.84-1.22, *P* = 0.89). The similar results were obtained in the stratified analyses by ethnicity, source of controls (population-based), and quality score of studies (Fig. 2, Table 3). The risk was also not significantly altered for *MTHFR* C677T polymorphism and MM in overall comparisons (CT vs. CC, OR = 1.04, 95%CI = 0.93-1.17, *P* = 0.49; TT vs. CC, OR = 1.16, 95%CI = 0.98-1.37, *P* = 0.08; TT vs. CC + CT, OR = 1.13, 95%CI = 0.98-1.32, *P* = 0.10; CT + TT vs. CC, OR = 1.07, 95%CI = 0.96-1.20, *P* = 0.22). In the subgroup analyses according to ethnicity, no significant association was observed in both Caucasians and Asians (Fig. 3, Table 3). However, when stratified by the quality score of studies (high and intermediate), a significantly increased risk for *MTHFR* C677T polymorphism and MM was detected in studies with intermediate quality (CT vs. CC, OR = 1.53, 95%CI = 1.03-2.27, *P* = 0.04; TT vs. CC, OR = 2.45, 95%CI = 1.36-4.43, *P* = 0.003; TT vs. CC + CT, OR = 1.94, 95%CI = 1.13-3.31, *P* = 0.02; CT + TT vs. CC, OR = 1.67, 95%CI = 1.15-2.44, *P* = 0.007) (Table 3).

### Publication bias and sensitivity analysis

The publication bias was detected using funnel plot and the results showed that there was no obvious asymmetry in the funnel plots, suggesting the absence of publication bias in the overall meta-analysis. We also assessed the stability of the overall results by sequential omission of individual studies. The result of sensitive analysis showed that no individual study could significantly influence the combined results, indicating the reliability and stability of our results.

## Discussion

MTHFR plays an important role in the regulation of DNA synthesis, methylation, and repair. Thus, DNA methylation and synthesis may be affected by alterations in the enzyme activity of MTHFR, which subsequently increases the incidence of malignancies^18^,^34^. Previous studies have confirmed that these common functional polymorphisms of *MTHFR* give rise to a thermolabile enzyme with significantly reduced enzyme activity^19^,^20^. Numerous studies have been conducted to investigate the relationship between *MTHFR* C677T or A1298C polymorphisms and the cancers risk, and these polymorphisms were associated with a low risk of colorectal cancer^35^, and an increased risk for non-Hodgkin lymphoma^36^. However, there are controversial findings about the role of *MTHFR* C677T and A1298C polymorphisms in the development of MM. González Ordóñez *et al.*^25^ showed that the 677CC genotype of *MTHFR* gene could be an effective protective factor against MM. Hatzimichael *et al.*^27^ did not observe significant difference in genotype distribution of *MTHFR* A1298C polymorphism between MM patients and controls. No significant association between *MTHFR* C677T or A1298C polymorphisms and MM susceptibility was found in Chiusolo *et al.*^24^, suggesting that variant alleles might not play a vital role in the development risk of MM.

To quantitatively and comprehensively evaluate the effect of *MTHFR* C677T and A1298C polymorphisms on MM risk, a meta-analysis including 10 case-control studies was performed. The present meta-analysis suggested that there was no significant association between *MTHFR* C677T or A1298C polymorphism and MM risk in overall comparisons and subgroup analyses by ethnicity and source of controls. Therefore, the extensively investigated C677T and A1298C functional polymorphisms in *MTHFR* may not play a crucial role in the etiology of MM, which was consistent with the study reported by Martino *et al.*^32^ The results of large-scale study with high statistical power clarified that none of the previously reported single-nucleotide polymorphisms, which were identified to be associated with genetic susceptibility to MM in the last years, were significantly associated with MM risk with the exception of one polymorphism in women, and none of the meta-analyses showed any significant association with MM risk including *MTHFR* C677T polymorphism^32^. However, the study carried out by Martino *et al.*^32^ did not provide any data about the meta-analysis and did not synthetically evaluate the association of *MTHFR* A1298C polymorphism with MM risk. In addition, the stratification analyses by quality score of studies found that C677T polymorphism in *MTHFR* was significantly associated with an increased risk for MM under all four genetic models in studies with intermediate quality, but not in high-quality studies, which suggested that the methodological quality of the included studies might be a critical effect factor on the association.

However, this meta-analysis has some limitations which need to be addressed. Our analyses were based on unadjusted OR values without adjustment for other covariates such as age, gender, folate intake status, and exposures, which may result in relatively low power to estimate the real association. Some stratification analyses might have insufficient statistical power to detect the effect because of the limited number of included studies. Folate status may influence the association of *MTHFR* polymorphisms with MM risk through gene-nutrition interaction. However, the potential gene-environment effect was not evaluated in this study due to the unavailability of original data.

In summary, the current meta-analysis found that *MTHFR* C677T and A1298C polymorphisms were not associated with the altered risk for MM. However, well-designed studies based on larger sample sizes are needed to validate the present findings.

## Materials and Methods

### Studies identification

Two authors independently conducted a systematic literature search in the PubMed, Elsevier, China National Knowledge Infrastructure platform and Wanfang databases to identify studies about the relationship between *MTHFR* C677T or A1298C polymorphisms and MM risk (up to December 20, 2014). The search terms and keywords used were as follows: “methylenetetrahydrofolate reductase” or “*MTHFR*”, “polymorphism” or “variation” or “variant” or “mutant”, and “multiple myeloma” or “MM” or “plasma cell myeloma” or “myeloma” or “myelomatosis”, without any restriction on the language. A manual search for references cited in the eligible articles was also performed to look for additional studies.

### Inclusion criteria

Studies included in this meta-analysis had to meet the following criteria: (a) case-control studies about the association of *MTHFR* C677T or A1298C polymorphisms with MM risk; (b) the case group had confirmed diagnosis; (c) genotype frequencies for both cases and controls were available; (d) the distribution of genotypes in the control group was in consistent with Hardy-Weinberg equilibrium (HWE). If there were multiple articles from the same study, the most relevant was included. The case reports, letters, meta-analysis, and reviews were excluded.

### Data extraction

The following information were extracted from each included study: first author’s name, publication year, country, ethnicity of the study population, source of controls, genotyping methods, sample size of cases and controls, genotypes distribution of the *MTHFR* C677T and A1298C polymorphisms in cases and controls, and HWE of control group. Two authors independently extracted information and disagreement was addressed by discussion between them.

### Quality assessment

The Newcastle-Ottawa Scale (NOS) was applied to assess the quality of the included studies independently by two reviewers^37^. The NOS includes three parameters of quality for case-control studies: selection of the study population, comparability of subjects, and exposure assessment. This scale, with a maximum score of 9 points, assigns 4 points for selection, 2 for comparability, and 3 for exposure. NOS scores of 7–9, 4–6, and 1–3 were considered as high, intermediate, and low-quality studies, respectively. Any discrepancies were addressed by re-evaluation of the original studies.

### Statistical analysis

The strength of association of *MTHFR* C677T and A1298C polymorphisms with MM risk was assessed by odds ratios (ORs) with 95% confidence intervals (CIs) under the heterozygote model (C677T: CT vs. CC; A1298C: AC vs. AA), homozygote model (C677T: TT vs. CC; A1298C: CC vs. AA), recessive model (C677T: TT vs. CC + CT; A1298C: CC vs. AA + AC) and dominant model (C677T: CT + TT vs. CC; A1298C: AC + CC vs. AA). The *Z*-test was used to determine the significance of combined ORs. The heterogeneity between included studies was evaluated by the Q-test. If *P* > 0.05, indicating that there exists no significant heterogeneity, the fixed-effects model (Mantel-Haenszel) was selected to combine the data, otherwise, the random-effects model (DerSimonian-Laird) was applied. Subgroup analyses were performed according to ethnicity (Asians and Caucasians), source of controls (population-based and hospital-based), and quality score of studies (high and intermediate). The publication bias was detected using funnel plot and sensitivity analysis was performed by sequential omission of individual studies to assess the stability of results. HWE of genotypes distribution in the control group was checked by the *χ*^*2*^-test. All the tests were two-sided and *P* < 0.05 was considered as statistically significant. The data analyses were performed using the software STATA v12.0 (Stata Corporation, College Station, TX) and Review Manager v5.2 (The Cochrane Collaboration, Oxford, UK).

## Additional Information

**How to cite this article**: Ma, L.-M. *et al.* Meta-analysis of the association of *MTHFR* polymorphisms with multiple myeloma risk. *Sci. Rep.* **5**, 10735; doi: 10.1038/srep10735 (2015).

## Figures and Tables

**Table 1:** Main characteristics of studies included in the meta-analysis.

**First author**	**Year**	**Country**	**Ethnicity**	**Control source**	**Genotyping method**	***MTHFR*polymorphisms**	**Quality Score**
Chiusolo^24^	2006	Italy	Caucasian	PB	PCR-RFLP	C677T; A1298C	8
González Ordóñez^25^	2000	Spain	Caucasian	Unknown	PCR-RFLP	C677T	4
González-Fraile^26^	2002	Spain	Caucasian	PB	PCR-RFLP	C677T; A1298C	8
Hatzimichael^27^	2010	Greece	Caucasian	PB	PCR-RFLP	A1298C	7
Jiang^28^	2014	China	Asian	HB	Microarray	C677T; A1298C	6
Kim^29^	2007	Korea	Asian	PB	PCR-RFLP; Real-time PCR	C677T; A1298C	7
Lima^30^	2008	Brazil	Caucasian	PB	PCR-RFLP	C677T; A1298C	8
Lincz^31^	2003	Australia	Caucasian	PB	PCR-RFLP	C677T; A1298C	6
Martino^32^	2014	IMMEnSE*	Caucasian	HB, PB	TaqMan assay	C677T	8
Moon^33^	2007	Korea	Asian	PB	TaqMan assay	C677T; A1298C	8

PB, Population-based; HB, Hospital-based; PCR, polymerase chain reaction; RFLP, restriction fragment length polymorphism;

^*^Seven European countries in the context of the International Multiple Myeloma rESEarch (IMMEnSE) consortium, including Italy, Poland, Spain, France, Portugal, Hungary, and Denmark.

**Table 2:** The genotypes distribution of *MTHFR* C677T and A1298C polymorphisms in cases and controls.

	**Distribution of C677T genotypes**							**Distribution of A1298C genotypes**						
**First author**	**Case**			**Control**				**Case**			**Control**			
	**CC**	**CT**	**TT**	**CC**	**CT**	**TT**	**HWE**	**AA**	**AC**	**CC**	**AA**	**AC**	**CC**	**HWE**
Chiusolo^24^	31	44	25	36	45	19	0.46	48	44	8	37	50	13	0.54
González Ordóñez^25^	5	17	4	92	88	20	0.88							
González-Fraile^26^	31	48	11	38	32	9	0.57	39	55	13	34	35	10	0.83
Hatzimichael^27^								24	18	3	39	32	6	0.87
Jiang^28^	9	11	10	72	66	19	0.52	23	5	2	109	46	2	0.24
Kim^29^	58	80	35	540	863	297	0.13	116	51	6	1147	500	53	0.87
Lima^30^	52	57	14	92	79	17	0.99	79	33	11	127	49	12	0.02
Lincz^31^	38	44	8	145	133	21	0.20	29	43	9	124	139	31	0.38
Martino^32^	554	525	185	767	787	243	0.07							
Moon^33^	57	103	36	144	196	94	0.08	136	52	8	307	120	7	0.22

HWE, Hardy-Weinberg equilibrium.

**Table 3:** Results of meta-analysis for *MTHFR* C677T and A1298C polymorphisms with MM risk.

**Variables**	**No.**	**Sample size**	**Heterozygote model**			**Homozygote model**			**Recessive model**			**Dominant model**		
		**Case/Control**	**OR(95% CI)**	***P***	*P*_h_*	**OR(95% CI)**	***P***	*P*_h_*	**OR(95% CI)**	***P***	*P*_h_*	**OR(95% CI)**	***P***	*P*_h_*
**C677T**														
Overall	9	2092/4954	1.04(0.93–1.17)	0.49	0.06	1.16(0.98–1.37)	0.08	0.19	1.13(0.98–1.32)	0.10	0.27	1.07(0.96–1.20)	0.22	0.06
Ethnicity														
Asian	3	399/2291	1.07(0.83–1.38)	0.60	0.25	1.39(0.74–2.60)	0.30	0.04	1.35(0.71–2.55)	0.36	0.01	1.10(0.87–1.40)	0.42	0.20
Caucasian	6	1693/2663	1.28(0.94–1.74)	0.11	0.04	1.16(0.95–1.40)	0.14	0.46	1.15(0.96–1.37)	0.14	0.95	1.31(0.98–1.76)	0.07	0.04
Control source														
PB	6	772/2800	1.18(0.97–1.42)	0.09	0.38	1.19(0.92–1.54)	0.20	0.87	1.09(0.87–1.38)	0.45	0.71	1.18(0.99–1.42)	0.07	0.53
Score														
High	6	1946/4298	1.00(0.89–1.14)	0.95	0.15	1.10(0.92–1.30)	0.29	0.85	1.09(0.93–1.27)	0.29	0.73	1.03(0.91–1.15)	0.66	0.25
Intermediate	3	146/656	1.53(1.03–2.27)	0.04	0.20	2.45(1.36–4.43)	0.003	0.26	1.94(1.13–3.31)	0.02	0.24	1.67(1.15–2.44)	0.007	0.18
**A1298C**														
Overall	7	732/2841	0.99(0.82–1.20)	0.92	0.50	1.14(0.77–1.68)	0.51	0.27	1.10(0.76–1.59)	0.62	0.30	1.01(0.84–1.22)	0.89	0.52
Ethnicity														
Asian	3	399/2291	0.95(0.74–1.22)	0.70	0.47	1.72(0.94–3.14)	0.08	0.28	1.74(0.96–3.15)	0.07	0.24	1.01(0.80–1.28)	0.94	0.69
Caucasian	4	333/550	1.05(0.78–1.42)	0.75	0.30	0.89(0.54–1.46)	0.65	0.48	0.86(0.54–1.37)	0.53	0.80	1.02(0.76–1.36)	0.91	0.22
Control source														
PB	6	702/2684	1.02(0.84–1.24)	0.86	0.59	1.09(0.73–1.61)	0.68	0.34	1.05(0.72–1.53)	0.82	0.46	1.03(0.85–1.24)	0.75	0.48
Score														
High	5	621/2390	0.98(0.79–1.21)	0.82	0.62	1.05(0.67–1.64)	0.83	0.23	1.04(0.68–1.61)	0.85	0.32	0.99(0.81–1.21)	0.93	0.48
Intermediate	2	111/451	1.06(0.67–1.68)	0.80	0.11	1.48(0.68–3.19)	0.32	0.23	1.27(0.62–2.62)	0.51	0.13	1.12(0.72–1.73)	0.62	0.23

OR, odds ratio; CI, confidence interval; *MTHFR*, methylenetetrahydrofolate reductase; MM, multiple myeloma; PB, Population-based; *P*_h_ value used to test the heterogeneity;

^*^If *P*_h_ > 0.05, the fixed-effects model was applied to combine the data. Otherwise, the random-effects model was selected.
